# HIV control through a single nucleotide on the HLA-I locus

**DOI:** 10.1186/1742-4690-9-S2-O47

**Published:** 2012-09-13

**Authors:** H Kløverpris, M Harndahl, J Carlson, A Leslie, M van der Stok, G Huang, F Chen, L Riddell, D Steyn, D Goedhals, C van Vuuren, J Frater, B Walker, T Ndung'u', S Buus, P Goulder

**Affiliations:** 1 University of Oxford, Oxford, UK; 2 University of Copenhagen, Copenhagen, Denmark; 3 University of KwaZulu-Natal, South Africa; 4 Royal Berkshire Hospital, Reading, UK; 5 Northampton General Hospital, UK; 6 University of Free State, Bloemfontein, South Africa; 7 Ragon Institute of MGH, MIT and Harvard, Boston, USA

## Background

In correlative studies HLA class I type is consistently found to have the strongest impact on HIV disease progression. However, the exact mechanism involved is complicated by several factors; many alleles are ligands for NK cells as well as CD8 T-cells, and strong linkage disequilibrium between Class I alleles makes it difficult to distinguish the effect of individual alleles from other HLAs or from other important loci found on the HLA haplotype, such as the recently described -35 SNP.

## Methods

Here we study two recently diverged HLA alleles, B*4201 and B*4202, which only differ by a single amino acid. Crucially, they occur primarily on identical Class I haplotypes and do not act as NK cell ligands. Therefore, they represent a unique opportunity to study the impact of a single HLA allele on HIV immune control not confounded by other genetic factors in a large outbred cohort (n=2,093) of C-clade infected individuals.

## Results

Here we show that the amino acid change in position 9 of the HLA-B molecule, is critical for peptide binding and significantly alters the Gag CTL epitopes targeted (P=2x10^-^10), measured both directly ex-vivo by ELISPOT and indirectly through CTL escape mutation (P=2x10^-8^). Strikingly, HLA-B*4201 is associated with significantly lower viral load setpoint than HLA-B*4202 (P=0.02).

## Conclusion

This naturally controlled experiment represents perhaps the clearest demonstration of the direct impact of particular HIV Gag specific CTL on disease control.

